# Supplementary material for: Elucidation of Regulatory Modes for Five Two-Component Systems in Escherichia coli Reveals Novel Relationships
Source: mSystems. 2020 Nov 10;5(6):e00980-20. doi: 10.1128/mSystems.00980-20 (PMC7657598; doi:10.1128/mSystems.00980-20)
Supplement: TABLE S1 [file mSystems.00980-20-st001.pdf]

Oligo Name	TCS Gene Name	sequence 5'- 3'
BaeR myc F	BaeR	TTTATCCGCGCCGTTTATGGCGTCGGTTACCGCTGGGAAGCCGACGCCTGCCGCATCGTTGTCGGATCCAGTCTTCGTGAT
BaeR myc R	BaeR	ATAATGTTGCCGGATCTGGTCTGAACGCCATCTCCGGCTAACAAAATAATGTCGCTAAAAAATTCCGGGGATCCGTCGACC
BaeR KO F	BaeR	GGGGTAAGCATTACAGTAGAGTTACCGCTGGAACGGGATTTACAGAGAGAAGTatgACCGattccggggatccgtcgacc
BaeR KO R	BaeR	GTTGCCGGATCTGGTCTGAACGCCATCTCCGGCTAACAAAATAATGTCGCTAAAActaAAgtgtaggctggagctgcttc
KdpE myc F	KdpE	GATCCCGCCCGCCCACGCCATTTCATTACTGAAACCGGTATTGGCTATCGGTTTATGCTTGTCGGATCCAGTCTTCGTGAT
KdpE myc R	KdpE	AAGCCGTATTTATTATTACGGCTTTAATTAATAAAAGGCAGGCTGTATTAAAATTAATATAATTCCGGGGATCCGTCGACC
KdpE KO F	KdpE	ACACTTCCCCAGCAAACTGCCCCTGAACTTGAAGAATTTCATGAGGATATgtgACAAACGattccggggatccgtcgacc
KdpE KO R	KdpE	TATtcaAAGCATAAACCGATAGCCAATACCGGTTTCAGTAATGAAATGGCGTGGGCGGGCgtgtaggctggagctgcttc
CpxR myc F	CpxR	AAAGATGGTCACCCGTGGTTTAAAACCTTGCGTGGTCGCGGCTATCTGATGGTTTCTGCTGTCGGATCCAGTCTTCGTGAT
CpxR myc R	CpxR	CCAGCGCCAGCGTCAGCCAGAAGATGGCGAAGATGCGCGCGGTTAAGCTGCCTAtcaTGAAATTCCGGGGATCCGTCGACC
CpxR KO F	CpxR	GTAAAGTCATGGATTAGCGACGTCTGATGACGTAATTTCTGCCTCGGAGGTATTTAAACAattccggggatccgtcgacc
CpxR KO R	CpxR	CACCAGCGCCAGCGTCAGCCAGAAGATGGCGAAGATGCGCGCGGTTAAGCTGCCTAtcaTgtgtaggctggagctgcttc
PhoB F myc	PhoB	GGGCATGACCGCATGGTGCAGACCGTGCGCGGTACAGGATATCGTTTTTCAACCCGCTTTGTCGGATCCAGTCTTCGTGAT
PhoB myc R	PhoB	CACGTAAGATACTCCAGTTAAGAAATCATAAGCCCTGCTCTGCGTCCGATGAGCAAGGCGAATTCCGGGGATCCGTCGACC
PhoB KO F	PhoB	TCTGACGCATAATGACGTCGCATTAATGATCGCAACCTATTTATTACAACAGGGCAAATCattccggggatccgtcgacc
PhoB KO R	PhoB	CACGTAAGATACTCCAGTTAAGAAATCATAAGCCCTGCTCTGCGTCCGATGAGCAAGGCGgtgtaggctggagctgcttc
ZraR F myc	ZraR	ACCGAAGCCGCCCGTCAGTTAGGGATCACGCGCAAAACGCTATTGGCAAAACTGTCGCGTGTCGGATCCAGTCTTCGTGAT
ZraR myc R	ZraR	CTGGGACGACTGCTTCTGCCGGAAAGATATCGGCTGGCGCGCTATCGAACGCGAGCAGAAAATTCCGGGGATCCGTCGACC
ZraR KO F	ZraR	CTCAACGTTCACCCTCTGGCTTCCGGTCAATATTACGCGTAAGGACCCACAAGGAtgaCGattccggggatccgtcgacc
ZraR KO R	ZraR	GGACGACTGCTTCTGCCGGAAAGATATCGGCTGGCGCGCTATCGAACGCGAGCAGAACTAgtgtaggctggagctgcttc
