# Supplementary figures and images for: Elucidation of Regulatory Modes for Five Two-Component Systems in Escherichia coli Reveals Novel Relationships
Source: mSystems. 2020 Nov 10;5(6):e00980-20. doi: 10.1128/mSystems.00980-20 (PMC7657598; doi:10.1128/mSystems.00980-20)

# BaeR

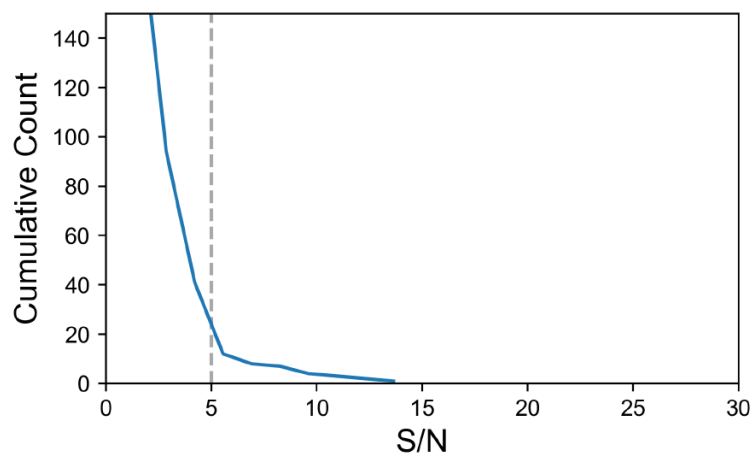

# CpxR

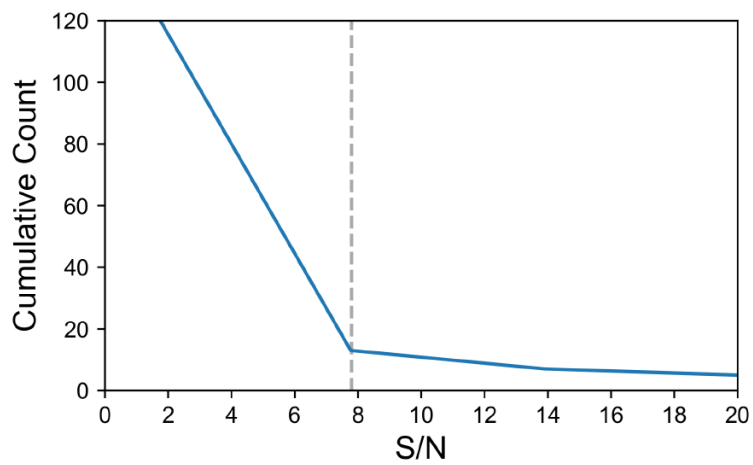

# KdpE

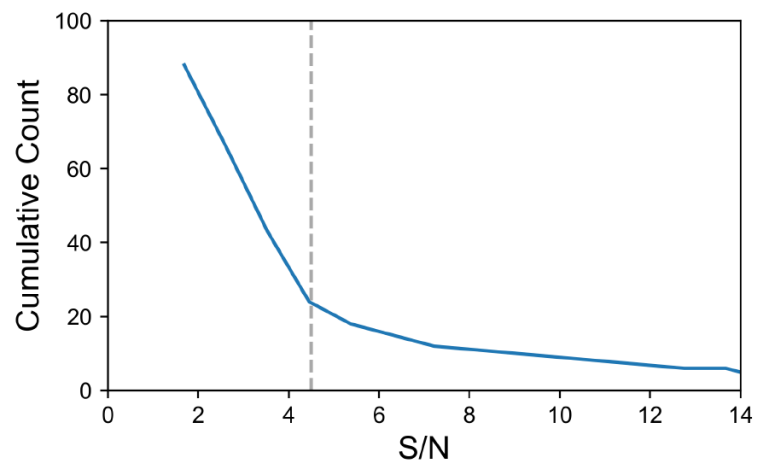

# PhoB

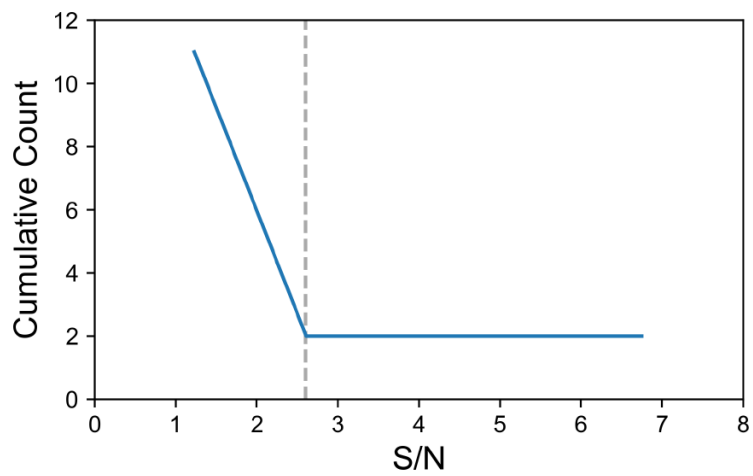

# ZraR

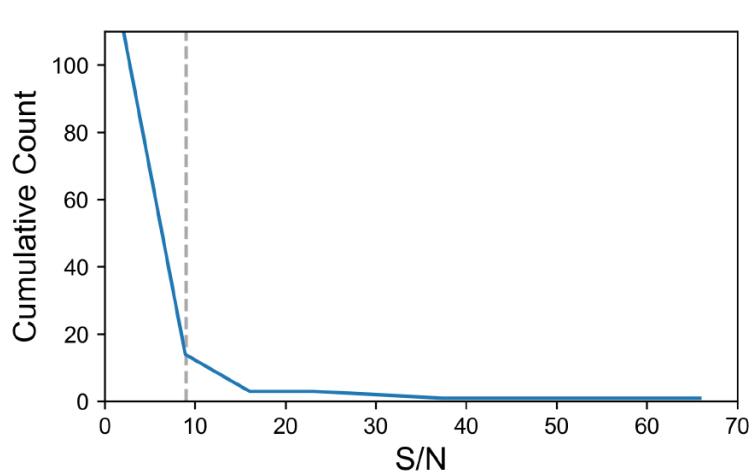

Supplement: FIG S1 [file mSystems.00980-20-sf001.pdf]

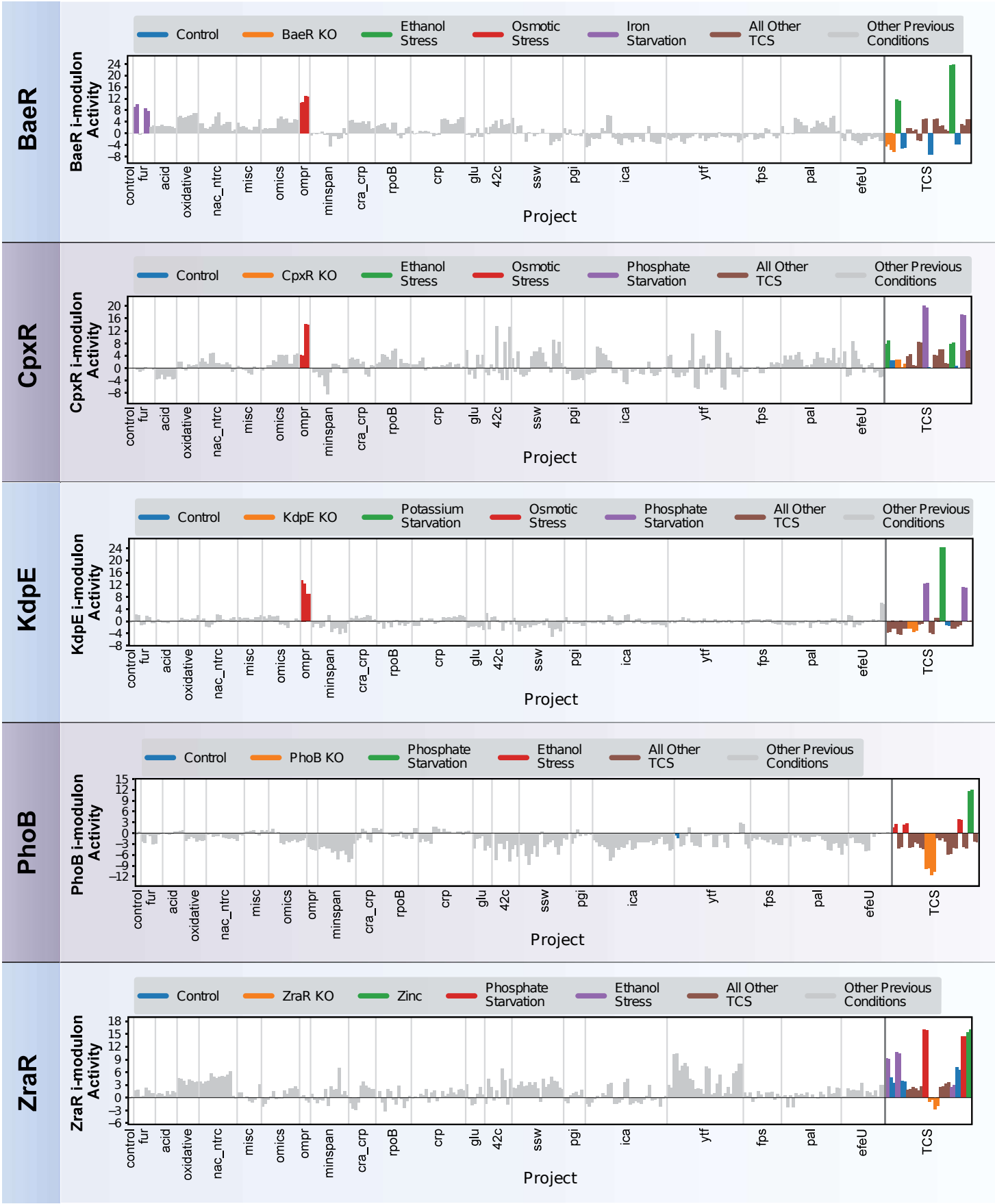

Supplement: FIG S2 [file mSystems.00980-20-sf002.pdf]
